# The hidden heat penalty of speeding up football

**DOI:** 10.1113/EP094149

**Published:** 2026-07-22

**Authors:** Edward T. Ashworth

**Affiliations:** ^1^ University of Basel Allschwil Switzerland

Hydration breaks are the most obvious change to the rules for the 2026 World Cup as part of an aim to protect players from the heat. However, a series of additional rule changes that have received far less attention are set to increase players’ heat exposure. These are the rules to speed up the game, including the eight‐second goalkeeper rule, faster restarts and the clampdown on time‐wasting (IFAB, 2025). The effects of how these changes together could actually increase risk have been minimally evaluated so far.

Playing football in the heat is highly demanding. Heat stress in sport is measured by the wet‐bulb globe temperature (WBGT), an index that combines air temperature, humidity and radiant heat. In games above 28°C WBGT, core temperatures can exceed 39.5°C, which lowers aerobic and cognitive performance (Mohr et al., 2012; Ozgunen et al., 2010). To mitigate such a rise in core temperature, players adjust behaviourally by reducing the amount of sprinting and high‐intensity running by roughly 8–10% (Carmo et al., 2026; Mohr et al., 2012; Schwarz et al., 2025).

At its simplest, a player's core temperature is a balance between the heat their muscles make and the heat they shed. This is easy to see with simple modelling (Ashworth, 2026). With the ball in play the average player produces around 1750 W of heat, whereas when the ball is out of play and the player is walking, they produce around 240 W, roughly seven times less. During the game, heat losses will range between around 600 and 850 W, meaning there is a net heat gain while the ball is in play, and a net heat loss when the ball is out of play.

This is how the new laws unintentionally contribute to heat load. Previous analyses of elite matches (Siegle & Lames, 2012) have shown the ball to be in play for a little under 60% of the game time. In the heat, under the old rules, the game slows further as stoppages can be lengthened by players, as seen in other sports (Morante et al., 2007; Périard et al., 2014), causing realised playing time to fall towards 50%. In contrast, applying the new laws prevents this, likely resulting in the ball being in play for around 70% of the match even in the heat (Ashworth, 2026). Across a 90‐min match that can amount to 15–20 more minutes of running, roughly 25% more total work, and consequently more heat production. At a WBGT of 28°C, a player able to slow the game by drawing out stoppages, could finish a match near 38.6°C, whereas the new rules would push them to near 39.6°C, a degree higher (Ashworth, 2026). At the WBGT 32°C limit for play, while following the new rules to speed the game up, players would reach core temperatures around 40°C, a level commonly associated with exertional heat illness.

To prevent this, the hydration break lets players cool down at the midpoint of each half. The use of chilled fluid and an ice towel during the break has been shown to lower the final core temperature by 0.39°C at WBGT 32°C compared to no break (Brown et al., 2024). In the sped‐up game this late‐match benefit may be smaller than the trial suggests, because a player who has just been cooled can work harder, and harder work makes more heat. A lower core temperature also gives a weaker drive for heat loss, so heat is shed more slowly. Both effects push the cooled player's core temperature back up and accelerate it towards the level it would be at if no hydration break had occurred. This is the main point of the break. It buys back the thermal room players need to sustain a higher intensity for a larger part of the match, which is exactly what fans want to see. The break still helps. Over a 90‐min match at WBGT 32°C, it can lower the peak core temperature by about 0.4°C and cuts the time spent above 39.5°C from about 34 min to about 2 min (Ashworth, 2026). As the time at the highest core temperatures is reduced, the overall cardiovascular stress is also reduced. While the heat stress imposed on players in hot conditions is likely more severe than before this World Cup, the integrity of the game is conserved even under such conditions. However, this benefit assumes optimal cooling, and should these rules be applied to lower levels of the game, the difference in access to cooling resources would also need to be considered.

Within this there are also further individual factors that make some players more at risk. The clearest is body size. A larger body produces more heat but has proportionally less skin to lose it through, so the heaviest players, around 98 kg, likely reach 40°C at the WBGT 32°C limit, the typical player around 39.7°C, and the lightest, around 68 kg, around 39.6°C, if matched for energy expenditure (Friesen et al., 2014). Sweat rate and heat‐acclimation status differ between players and influence how effectively they shed heat (Kurdak et al., 2010), while environmental conditions, especially humidity, play a major role by limiting evaporation so the core temperature rises faster for everyone.

Overall, in genuinely hot conditions, the hydration breaks serve as a method to ensure that the intensity and quality of play can be maintained. While this may seem to elevate core temperature compared to the prior rules, the prevention of sustained time at higher core temperatures strongly mitigates the risks.

## AUTHOR CONTRIBUTIONS

Sole author.

## CONFLICT OF INTEREST

None declared.

## FUNDING INFORMATION

None.

## GENERATIVE AI STATEMENT

Generative AI was not used in preparing this article.
